# Selective Internal Radiation Combined with Chemotherapy Maintains the Quality of Life in Intrahepatic Cholangiocarcinomas

**DOI:** 10.3390/curroncol28060384

**Published:** 2021-11-08

**Authors:** Camille Goislard de Monsabert, Yann Touchefeu, Boris Guiu, Boris Campillo-Gimenez, Olivier Farges, David Tougeron, Isabelle Baumgaertner, Ahmet Ayav, Luc Beuzit, Marc Pracht, Astrid Lièvre, Samuel Le Sourd, Karim Boudjema, Yan Rolland, Etienne Garin, Eveline Boucher, Julien Edeline

**Affiliations:** 1 Medical Oncology, Centre Eugène Marquis, 35000 Rennes, France; c.goislard@rennes.unicancer.fr (C.G.d.M.); b.campillo@rennes.unicancer.fr (B.C.-G.); m.pracht@rennes.unicancer.fr (M.P.); s.lesourd@rennes.unicancer.fr (S.L.S.); Evelyne.Boucher@bsci.com (E.B.); 2 Centre Hospitalo-Universitaire Nantes, 44000 Nantes, France; yann.touchefeu@chu-nantes.fr; 3 Centre Hospitalo-Universitaire Montpellier, 34000 Montpellier, France; b-guiu@chu-montpellier.fr; 4 Laboratoire du Traitement du Signal et de l’Image U1099, Centre Hospitalo-Universitaire, Université Rennes 1, F-35000 Rennes, France; 5 Centre Hospitalo-Universitaire Beaujon, 92100 Clichy, France; olivier.farges@bjn.aphp.fr; 6 Centre Hospitalo-Universitaire Poitiers, 86000 Poitiers, France; david.tougeron@chu-poitiers.fr; 7 Centre Hospitalo-Universitaire Mondor, 94000 Créteil, France; isabelle.baumgaertner@aphp.fr; 8 Centre Hospitalo-Universitaire Nancy, 54000 Nancy, France; a.ayav@chru-nancy.fr; 9 Radiology, Centre Hospitalo-Universitaire Pontchaillou, 35000 Rennes, France; luc.beuzit@chu-rennes.fr; 10 Gastroenterology, Centre Hospitalo-Universitaire Pontchaillou, 35000 Rennes, France; astrid.lievre@chu-rennes.fr; 11 Inserm, Centre Eugène Marquis, COSS (Chemistry Oncogenesis Stress Signaling)-UMR1242, Université Rennes, F-35000 Rennes, France; 12 Hepatobiliary Surgery, Centre Hospitalo-Universitaire Pontchaillou, 35000 Rennes, France; karim.boudjema@chu-rennes.fr; 13 Radiology, Centre Eugène Marquis, 35000 Rennes, France; y.rolland@rennes.unicancer.fr; 14 Nuclear Medicine, Centre Eugène Marquis, 35000 Rennes, France; e.garin@rennes.unicancer.fr; 15 Institut national de la rechercher médicale, Institut national de la recherche agronomique, Centre de Lutte contre le Cancer Eugène Marquis, Institut NUMECAN (Nutrition Metabolisms and Cancer), Université Rennes, F-35000 Rennes, France

**Keywords:** biliary tract cancer, radioembolization, Yttrium-90, chemotherapy, patient-reported outcomes

## Abstract

Background: In the Yttrium-90 Microspheres in Cholangiocarcinoma (MISPHEC) single-arm phase 2 trial, concomitant chemotherapy and selective internal radiotherapy (SIRT) showed antitumor activity as a first-line treatment of unresectable intrahepatic cholangiocarcinomas (ICCs). In this sub-analysis, we aimed to evaluate one of the secondary endpoints, the health-related quality of life (QoL), evaluated with an EORTC QLQ-C30 instrument at the baseline and during treatment. Methods: The MISPHEC trial included treatment-naïve patients with an unresectable ICC between November 2013 and June 2016. Patients received concomitant first-line chemotherapy with cisplatin and gemcitabine for 8 cycles; SIRT was administered during cycle 1 (for patients with unilobar disease) or cycles 1 and 3 (for patients with bilobar disease) using glass Yttrium-90 microspheres. We evaluated the QoL—measured by the QLQ-C30 questionnaire—at the baseline, every 8 weeks during chemotherapy and follow-up, between 12 and 15 weeks after embolization and every 12 weeks after a liver resection if applicable. Results: A total of 41 patients were included, of which 34 completed questionnaires at the baseline. No clinically significant changes in the global health score or the sub-scales of the QLQ-C30 were observed during follow-up. The physical, social and role function mean score worsened during treatment and fatigue, nausea and pain scores increased although the differences were not clinically significant. In patients undergoing subsequent surgery, the QoL was not impaired. Conclusions: A combination of SIRT and chemotherapy with gemcitabine and cisplatin as the first-line treatment of unresectable ICCs was found to maintain the QoL.

## 1. Introduction

The incidence of intrahepatic cholangiocarcinomas (ICCs) has been increasing in Western countries. For advanced ICCs, doublet chemotherapy with cisplatin and gemcitabine became the standard treatment after the ABC-02 trial [1] reported a median overall survival (OS) of 11.7 months, confirmed by a meta-analysis [2,3]. However, very limited data exist regarding quality of life (QoL) in this context [4,5,6,7,8].

Selective internal radiotherapy (SIRT) using Yttrium-90 (90Y)-labeled microspheres, also known as radioembolization, is applied as a locoregional treatment for both primary liver malignancies and hepatic metastases. The QoL of patients treated with SIRT has been mainly studied in the context of HCCs or metastases but never in the context of ICCs [9,10,11,12,13,14,15]. To correctly evaluate the benefit-over-risk ratio of the treatment from the perspective of a patient, the QoL endpoints are of paramount importance.

The Yttrium-90 Microspheres in Cholangiocarcinoma (MISPHEC) trial [16] was a prospective multi-center single-arm phase 2 trial that studied SIRT combined with chemotherapy in the first-line treatment of unresectable locally advanced (also including limited extra-hepatic disease) ICCs (the results were previously published). A total of 41 patients were treated between November 2013 and June 2016 with a response rate by RECIST of 39% and a high disease control rate at 3 months of 98%. The median OS was 22 months and the progression-free survival (PFS) was 14 months. A high proportion of patients were down-staged to surgical intervention and had favorable post-surgical outcomes. The results of other studies confirmed the promising outcomes with the locoregional treatment of ICCs; however, this should be confirmed in randomized studies [17].

In this study, we aimed to evaluate the effects of the combination of SIRT and chemotherapy with cisplatin and gemcitabine on the QoL measured through the EORTC QLQ-C30 questionnaire of patients treated in the MISPHEC trial.

## 2. Methods

### 2.1. Study Design and Population

The Yttrium-90 Microspheres in Cholangiocarcinoma (MISPHEC) trial was a first-line multi-center open-label single-arm phase 2 clinical trial. The efficacy and safety results have been reported previously [16].

Briefly, patients were eligible if they had a liver-dominant unresectable ICC.

The trial was conducted across 7 centers in France from 12 November 2013 to 21 June 2016. The trial was approved by Comité de protection des personnes Ouest V ethics committee, Rennes, France (6 September 2012, reference 12/19-852) and was conducted according to good clinical practice and the Declaration of Helsinki. All participants provided written informed consent before inclusion in the trial. The protocol was registered at clinicaltrials.gov (NCT01912053).

### 2.2. Procedures

After inclusion, the patients initiated chemotherapy with a gemcitabine plus cisplatin regimen. In the case of unilobar involvement, SIRT was performed during cycle 1 (days 3–21); in the case of bilobar involvement, a first SIRT procedure was performed as described previously and a second was performed during cycle 3 (days 3–21) to cover both lobes of the liver.

Chemotherapy was continued for a recommended number of 6 cycles but the prolongation of chemotherapy (biweekly gemcitabine plus cisplatin or gemcitabine alone) was accepted when deemed to be necessary by the investigators. Details of the treatment have been previously published [16].

### 2.3. Outcomes

The primary endpoint of the MISPHEC trial was a response rate (RR) according to the Response Evaluation Criteria in Solid Tumors (RECIST) 1.1 at 3 months, as reviewed by the investigators.

We evaluated the QoL with the QoL Questionnaire-Core 30 (QLQ-C30) version 3.0 [18]. The EORTC QLQ-C30 is a 30-item questionnaire that consists of five multi-item functioning scales (physical, role, cognitive, emotional and social), one multi-item global health status or QoL scale, three multi-item symptom scales (fatigue, pain and nausea or vomiting) and six single-item measures for dyspnea, loss of appetite, insomnia, constipation, diarrhea and perceived financial difficulties. Patients completed a paper-based questionnaire every 8 weeks during and after chemotherapy and between weeks 12 and 15 after radioembolization. In the case of subsequent surgery, the QoL was evaluated in the same way at the time of surgery and every 12 weeks thereafter.

We present here the means over time of the different items of the QLQ-C30 and a summary score based on the 13 scales (27 items) [19] in the overall population and according to the treatment received (1 vs. more SIRT; surgery vs. no surgery).

### 2.4. Statistical Analysis

The study was powered for the primary endpoint, which was the RR at 3 months. At least 41 patients were required to be included in the study. The final analysis included 41 treated patients. The analysis of the data of the QoL was a prespecified secondary endpoint.

All the scales and single-item measures of the QLQ-C30 ranged in score from 0 to 100. A high scale score represented a higher response level. A higher score for a functional scale represented a higher level of functioning and a higher score for the global health status represented a better QoL; conversely, a higher score for a symptom scale or an item represented a higher symptom burden [18]. According to Osaba et al., who compared the changes seen in the QoL scores against the Subjective Significance Questionnaire (SSQ), a 5 to 10 point change from the baseline (either deterioration or improvement) is a ‘little’ change, a 10 to 20 point change is a ‘moderate’ change and a ‘very large’ change corresponds with a change greater than 20 [20]. In addition, we used the QLQ-C30 summary score developed by Giesinger et al., which was calculated as the mean of the combined 13 QLQ-C30 scales and item scores (excluding the global QoL and the financial impact); a higher score indicated a better QoL [19].

We analyzed the overall population, then the sub-groups with either 1 or 2 SIRT sessions and finally the sub-group of resected patients. The latest available version of R statistical software was used for all analysis.

## 3. Results

### 3.1. Population

Between 12 November 2013 and 21 June 2016, 56 patients were screened and 41 were included in the analysis of the intent-to-treat population. Of the 41 patients included in the study, 26 (63%) were male with a mean age of 67 (range: 36–82) years. The characteristics of the population are reported in Table 1. The median follow-up was 36 months (95% CI, 26–51 months; range, 1–56 months).

### 3.2. Treatment

The median number of cycles of the chemotherapy delivered was 6 (range, 1–15 cycles) with a relative dose intensity of 81% for gemcitabine and a relative dose intensity of 88% for cisplatin. A total of 27 patients (65%) had 1 SIRT session, 12 (30%) had 2 sessions and 2 (5%) had 3 sessions (because of hepatic arterial anatomic features).

A total of 29 patients (71%) experienced grade 3 or 4 toxic effects, especially hematologic disorders (neutropenia (51%); thrombocytopenia (24%)). Asthenia was the most frequently reported grade 1–2 adverse event (78%) followed by anorexia (51%) and nausea (44%).

### 3.3. Global Health Score

**Figure 1:**
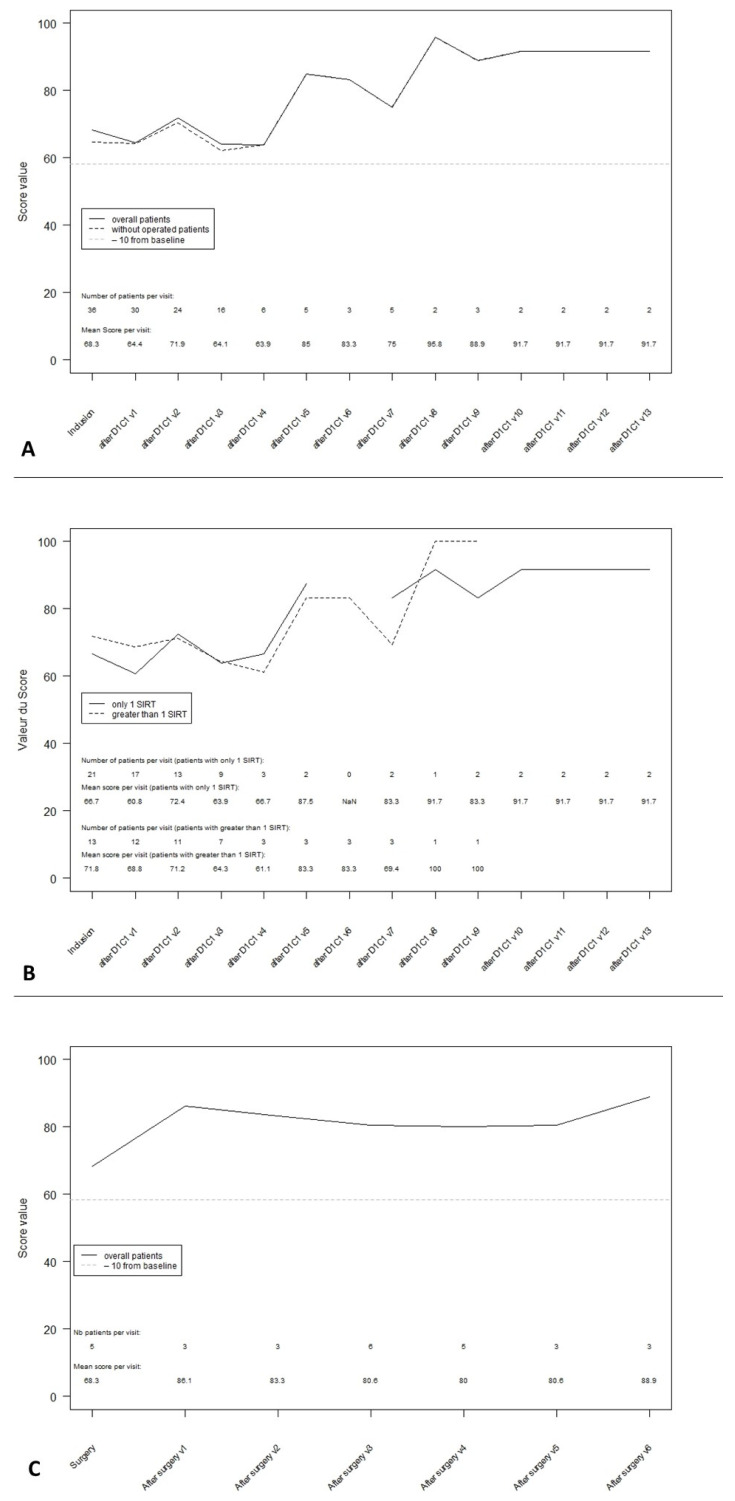
Global health status transformed score during follow-up. (**A**): Global health status score in ITT overall population. (**B**): Global health status score according to the number of SIRT procedures received. (**C**): Global health status score post-surgery.

At the baseline, 34 of the 41 patients completed these items of the QLQ-C30 questionnaire (85% of the included population). The mean global health score was 68.3. At week 8, after 2 cycles of chemotherapy and 1 or 2 SIRT sessions, the mean global health score was 64.4. During follow-up, no clinically significant decrease of the mean global health score was observed. The small amount of data after 48 weeks led to caution about the observed improvement in the QoL (Figure 1A).

At 24 weeks from the initiation of chemotherapy, the mean global health score was 63.9 after a single SIRT session vs. 66.7 at the baseline (*n* = 21) and 64.3 after 2 SIRT sessions vs. 71.8 at the baseline (*n* = 13). This 7.5 decrease suggested a small change only for patients with 2 SIRT sessions (Figure 1B).

After treatment, 9 patients (22%) were down-staged to surgical intervention. At the time of surgery, the global health was scored at 68.3 by 5 patients. The mean QoL after surgery is described in Figure 1C. Despite the short follow-up, there was no evidence of a deterioration in the QoL after surgery.

### 3.4. Functioning Scales

**Figure 2:**
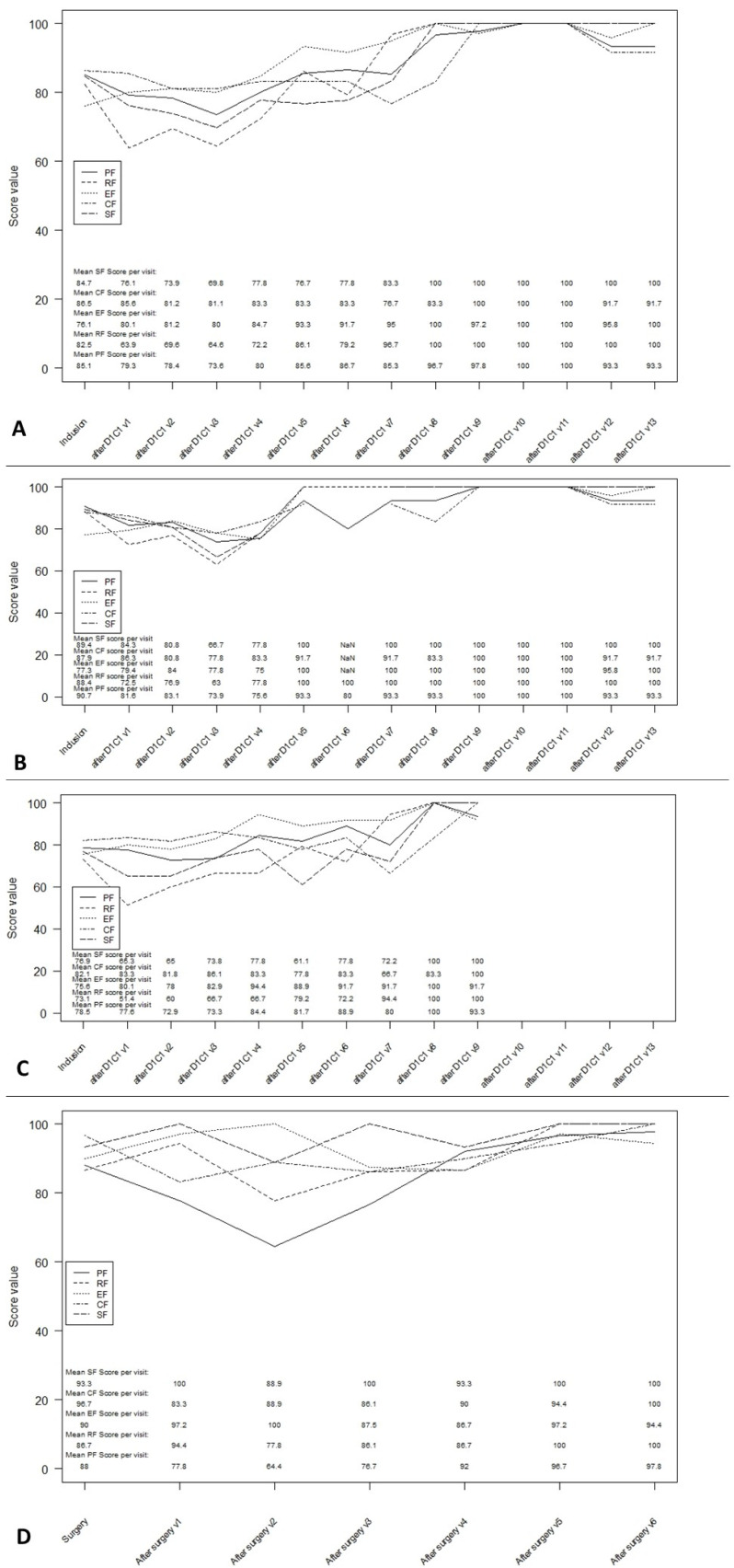
Multi-item functioning scales during follow-up. (**A**): Multi-item functioning score in ITT overall population. (**B**): Multi-item functioning score in patients with one SIRT procedures. (**C**): Multi-item functioning score in patients with more than one SIRT procedures. (**D**): Multi-item functioning score post-surgery. PF: physical functioning; RF: role functioning, EF: emotional functioning; CF: cognitive functioning; SF: social functioning.

A decrease of more than 10 points compared with the baseline score was observed at week 36 for the physical functioning scale, at week 24 for the social functioning scale and at week 12 for the role functioning scale. These scores all improved after week 36 but based on a few available data. There was no significant deterioration of cognitive and emotional functioning (Figure 2A).

These same scales were affected after one or more SIRT sessions up to week 36 and then improved (Figure 2B). After surgery, only the physical function score decreased at the second assessment, then improved to the baseline score (Figure 2C). The scores on the other scales did not vary significantly from the baseline.

### 3.5. Symptom Scales

**Figure 3:**
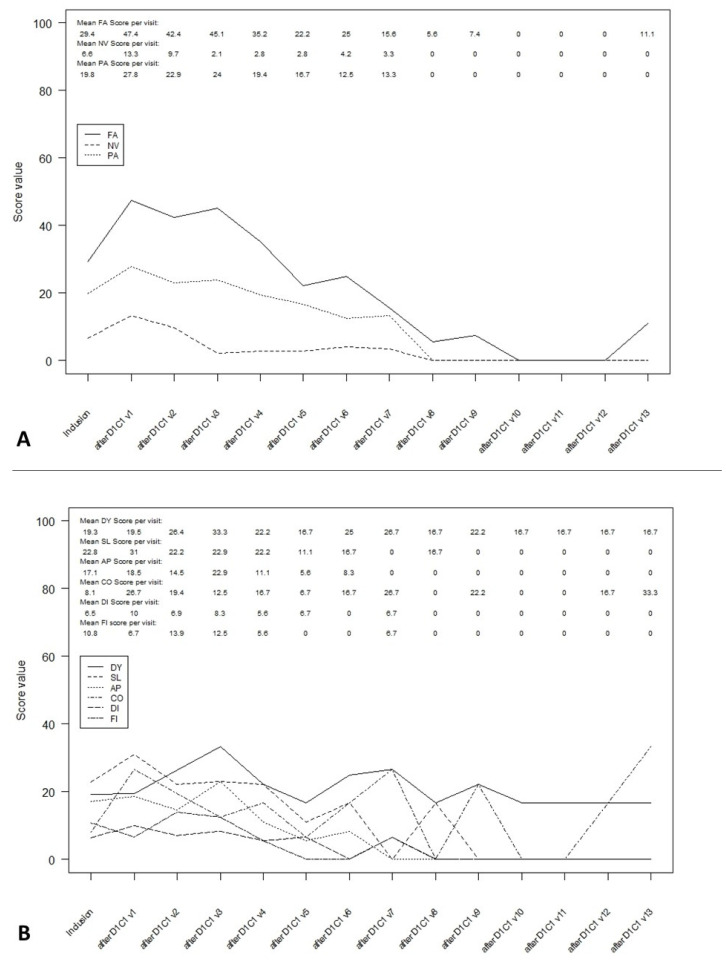
Symptom scales in ITT overall population during follow-up. (**A**): Multi-item symptom scales in ITT overall population. (**B**): Single-item symptom scales in ITT overall population. FA: fatigue; NV: Nausea and vomiting; PA: pain; DY: dyspnea; SL: insomnia; AP: appetite loss; CO: constipation; DI: diarrhea; FI: financial difficulties.

In the overall population, the fatigue, nausea and vomiting as well as the pain scores deteriorated at the first assessment during chemotherapy (Figure 3). The control of symptoms was better or even excellent after 48 weeks. The impact of the treatment on the constipation and insomnia scales was similar (deterioration at the first assessment and then improvement). The loss of appetite score and diarrhea score remained stable over time.

These results were similar after one or more SIRT sessions (Figure S1). After surgery, all symptoms were stable or controlled except for fatigue and pain, which deteriorated at the second evaluation before improving (Figure S2).

### 3.6. QLQ-C30 Summary Score

**Figure 4:**
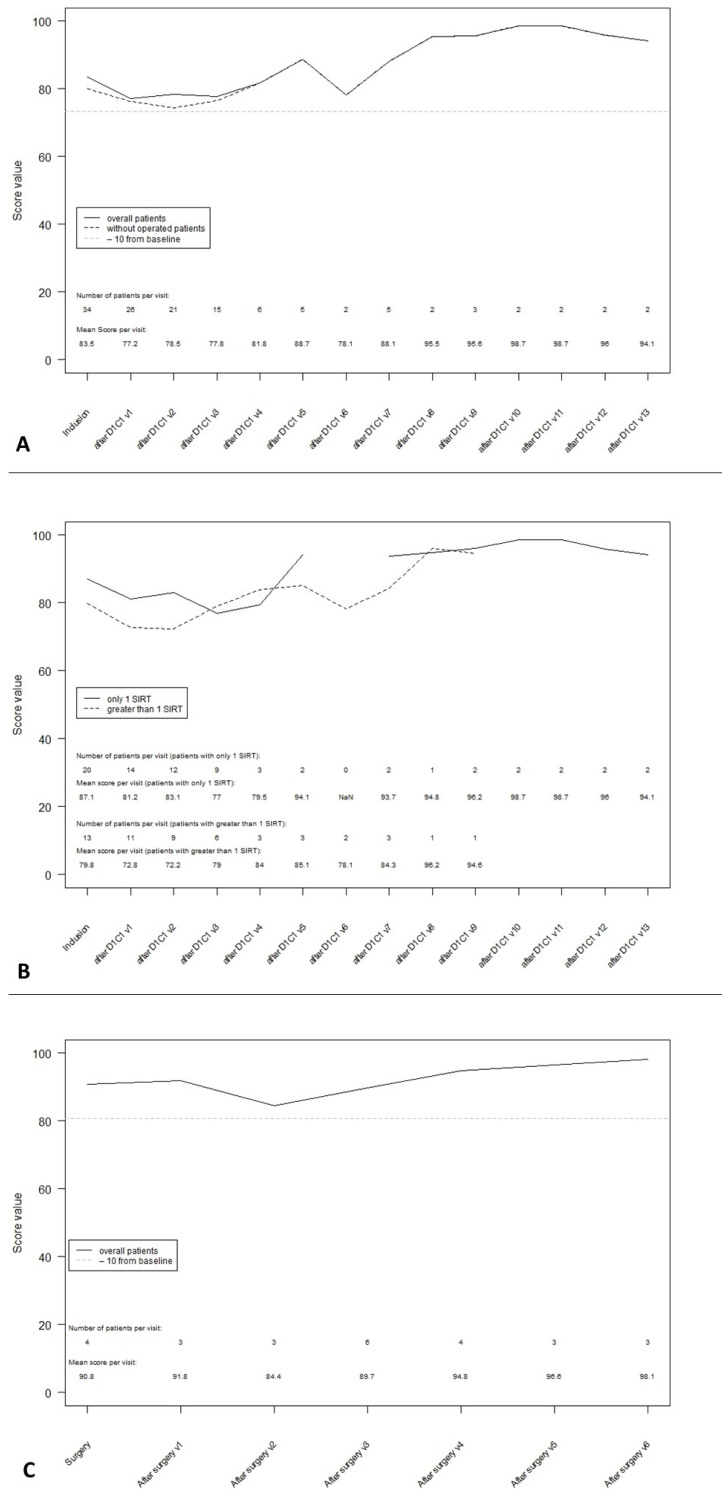
QLQ-C30 summary score during follow-up. (**A**): QLQ-C30 summary score in ITT overall population. (**B**): QLQ-C30 summary score according to the number of SIRT sessions received. (**C**): QLQ-C30 summary score post-surgery.

The baseline summary score was 83.5 based on the data from 34 patients. There was no significant change in this score during treatment (first assessment, score 77.2) or during follow-up (at 48 weeks, score 81.8) (Figure 4A). The QLQ-C30 summary score appeared to be better in patients with a single SIRT session than in patients with more than one SIRT session at the baseline (87.1 and 79.8 for 1 or more SIRT sessions, respectively), week 12 (81.2 and 72.8, respectively) and week 24 (83.1 and 72.2, respectively) (Figure 4B). In patients undergoing surgery, the summary score was stable over time (Figure 4C).

## 4. Discussion

The main result of this study was that despite significant adverse events reported in the MISPHEC trial (most of them being hematological), the QoL of the patients did not seem to be affected by the treatment.

The symptoms associated with tumor involvement in ICCs are rapidly disabling. Patients also face the side effects of the treatments, all of which may affect the QoL. Few studies have confirmed the benefit of treatment on the QoL for advanced cholangiocarcinomas, including palliative-intent chemotherapy [4,5,6,7,8]. Based on the ABC-02 study, a cisplatin and gemcitabine combination is the standard first-line chemotherapy regimen for patients with advanced biliary tract cancer [1]. The QoL analysis of the ABC-02 trial demonstrated an improvement in the cisplatin–gemcitabine arm vs. the gemcitabine arm and could, therefore, not be compared with our data [4].

A multiple single-center series reported the results of SIRT among patients with locally advanced ICCs [21,22,23,24,25,26,27,28,29]. Systematic reviews have suggested the activity of a SIRT treatment as well as other locoregional therapies [17,30,31]. The efficacy of SIRT has recently been confirmed with an acceptable safety profile in the prospective MISPHEC trial [16]. However, to the best of our knowledge, no publication to date has described the QoL with SIRT in ICCs. Locoregional therapies showed a benefit of the QoL in the treatment of primary hepatic malignancies, mainly hepatocellular carcinomas [9,10,11,12,13,14,15].

Our study is the first to report prospective QoL data in patients diagnosed with advanced ICCs treated with SIRT combined with doublet chemotherapy according to the ABC-02 protocol. The overall analysis of the global health score, functional scales and symptoms showed an initial small and non-clinically significant worsening that returned to the baseline level with a subsequent follow-up. Using the thresholds of clinical importance as defined by Giesinger et al. for the EORTC QoL group, only physical function was impaired from week 12 onwards [32]. The symptomatic scores were below the threshold. The scores for fatigue, nausea, vomiting and pain crossed the threshold at the first assessment but corrected later during follow-up. The QLQ-C30 summary score remained stable over time after one or two SIRT sessions. As the QoL is affected by both the treatment and disease, it was impossible to determine which of the results seen here depended on chemotherapy, SIRT and/or the disease.

Our analysis has a few limitations. The inclusion criteria were restrictive, related to aggressive strategy and treatment with a risk of toxicity. As the number of patients included was relatively low and because of the amount of missing data, the statistical power was limited and uncertainties remain with regard to the QoL data of this regimen. We did not use the EORTC QLQ-BIL21, the only disease-specific QoL questionnaire for patients with cholangiocarcinomas and gallbladder cancer, as it was not available at the time of the design of the MISPHEC trial [33]. The single-arm nature of the study limited the interpretation of the results, particularly as we lacked comparative data with cisplatin–gemcitabine chemotherapy. We could not conclude on the relative impact of chemotherapy or SIRT. The SIRT Followed by CIS-GEM Chemotherapy Vs. CIS-GEM Chemotherapy Alone as First Line Treatment of Patients With Unresectable Intrahepatic Cholangiocarcinoma (SIRCCA) phase 3 trial intended to confirm and refine the current data; however, the trial was closed prematurely due to a slow accrual.

## 5. Conclusions

These data indicate that the QoL was not clinically or meaningfully impaired by the combination of SIRT and cisplatin–gemcitabine chemotherapy as a first-line treatment for intrahepatic cholangiocarcinomas. It is possible that SIRT and chemotherapy may help to maintain the QoL of ICC patients although further studies are needed to confirm this.

## Figures and Tables

**Table 1:** Characteristics of the patients included.

Characteristics		All Included Patients (*n* = 41)	Down-Staged Patients (*n* = 9)
Median age at inclusion		67.3 (36.7–82.2)	71.2 (46.5–74.9)
Gender	Male	26 (63%)	4 (44%)
Cirrhosis		12 (29%)	2 (22%)
Child Pugh score at inclusion in patients with cirrhosis	A5	9 (75%)	2 (100%)
	A6	2 (16%)	0 (0%)
	B7	1 (8%)	0 (0%)
Performance status at inclusion (*n* = 40)	PS0	26 (65%)	7 (78%)
Albumin (g/L) (*n* = 39)		40 (24–47)	41 (39–44)
Prothrombin time (% relative to control)		89 (32–117)	90 (73–117)
Total bilirubin at inclusion (µmol/L)		13.3 (4–38)	13.6 (4–20.1)
ALT (UI/L)		28 (10–346)	20 (10–346)
AST (UI/L)		36 (12–138)	27 (12–115)
Alkaline phosphatase (UI/L)		111 (49–366)	106 (52–300)
Gamma GT rate (UI/L) (*n* = 40)		136.5 (25–613)	166 (61–597)
CA19.9 (*n* = 40)		52 (0.6–32099)	36.5 (1–499)
CEA (*n* = 40)		3.1 (0.4–51)	2.4 (1–5.1)
Previous resection		5 (12%)	0 (0%)
Days between diagnosis and enrollment		48 (13–728)	63 (14–77)
Unifocal tumor		14 (34%)	7 (78%)
Unilobar disease		27 (66%)	8 (89%)
Liver hilar lymph nodes ≤ 3 cm		12 (29%)	2 (22%)
Abdominal lymph nodes		14 (34%)	2 (22%)
Lung metastasis ≤ 1 cm		7 (17%)	0 (0%)
Patient with locally advanced disease only (including hilar nodules) without abdominal lymph nodes or lung metastasis		24 (58%)	7 (78%)

ALT: alanin transferase; AST: aspartate transferase; GT: glutamyl transferase; CA: carcino antigen; CEA: carcino-embryonic antigen.

## Data Availability

The data presented in this study are available on request from the corresponding author. The data are not publicly available due to sensitive clinical data involved.

## Supplementary Materials

The following are available online at https://www.mdpi.com/article/10.3390/curroncol28060384/s1, Figure S1: Symptom scales after one or more SIRT sessions, Figure S2: Symptom scales after surgery.
