# Supplementary material for: Selective Internal Radiation Combined with Chemotherapy Maintains the Quality of Life in Intrahepatic Cholangiocarcinomas
Source: Curr Oncol. 2021 Nov 8;28(6):4530–41. doi: 10.3390/curroncol28060384 (PMC8628701; doi:10.3390/curroncol28060384)
Supplement: Supplementary file 1 [file curroncol-28-00384-s001.zip › curroncol-1367745-supplementary.pdf]

Current Oncology

MDPI

Supplementary Materials

Selective Internal Radiation Combined with Chemotherapy Maintains the Quality of Life in Intrahepatic Cholangiocarcinomas

Camille Goislard de Monsabert, Yann Touchefeu, Boris Guiu, Boris Campillo-Gimenez, Olivier Farges, David Tougeron, Isabelle Baumgaertner, Ahmet Ayav, Luc Beuzit, Marc Pracht, Astrid Lièvre, Samuel Le Sourd, Karim Boudjema, Yan Rolland, Etienne Garin, Eveline Boucher and Julien Edeline

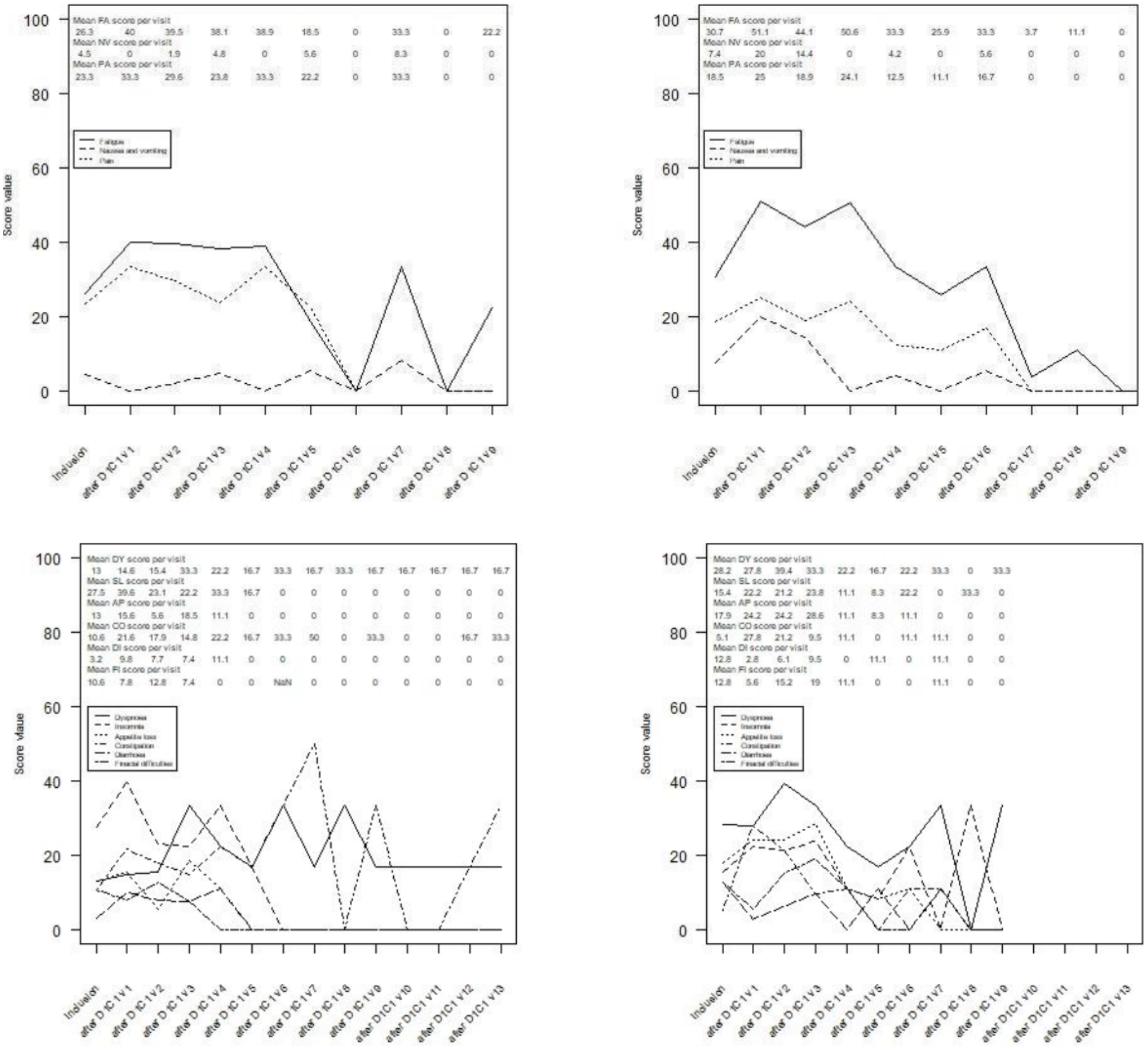

Figure S1. Symptom scales after one or more SIRT sessions.

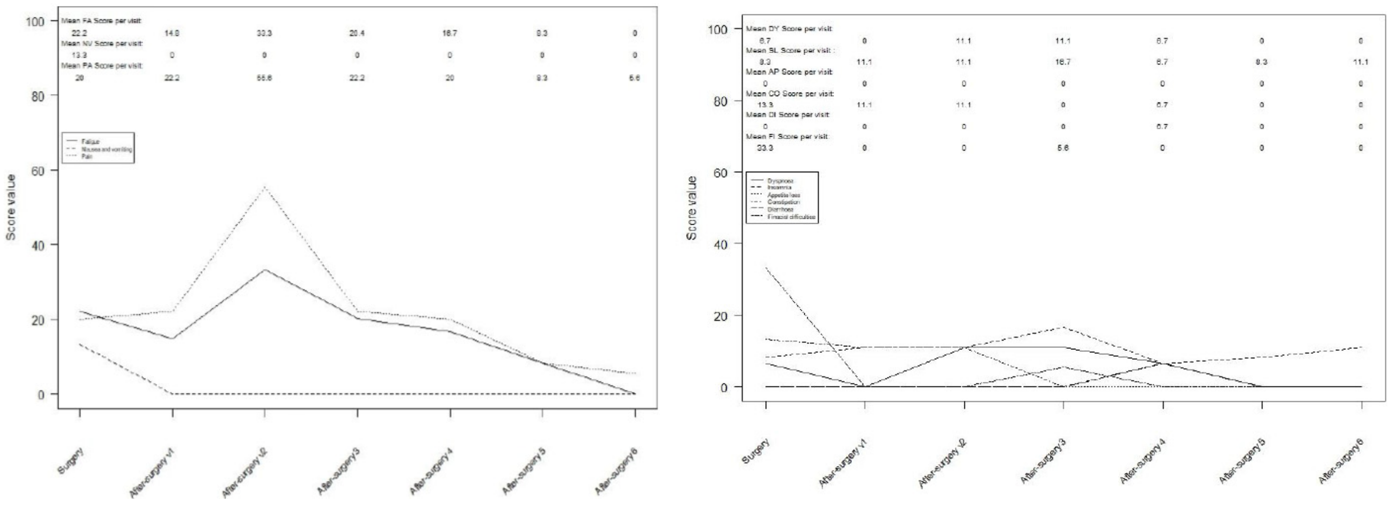

Figure S2. Symptom scales after surgery.

Copyright: © 2021 by the authors. Licensee MDPI, Basel, Switzerland. This article is an open access article distributed under the terms and conditions of the Creative Commons Attribution (CC BY) license (http://creativecommons.org/licenses/by/4.0/).
